# An electroencephalography connectome predictive model of major depressive disorder severity

**DOI:** 10.1038/s41598-022-10949-8

**Published:** 2022-04-26

**Authors:** Aya Kabbara, Gabriel Robert, Mohamad Khalil, Marc Verin, Pascal Benquet, Mahmoud Hassan

**Affiliations:** 1 Lebanese Association for Scientific Research, Tripoli, Lebanon; 2 MINDig, F-35000 Rennes, France; 3 Academic Department of Psychiatry, Centre Hospitalier Guillaume Régnier, Rennes, France; 4 Empenn, U1228, IRISA, UMR 6074, Rennes, France; 5 Comportement et Noyaux Gris Centraux, EA 4712, CHU Rennes, Université de Rennes 1, 35000 Rennes, France; 6 Azm Center for Research in Biotechnology and Its Applications, EDST, Tripoli, Lebanon; 7 CRSI Research Center, Faculty of Engineering, Lebanese University, Beirut, Lebanon; 8 Univ Rennes, Inserm, LTSI-U1099, F-35000 Rennes, France; 9 School of Science and Engineering, Reykjavik University, Reykjavik, Iceland

**Keywords:** Data processing, Depression, Magnetoencephalography

## Abstract

Emerging evidence showed that major depressive disorder (MDD) is associated with disruptions of brain structural and functional networks, rather than impairment of isolated brain region. Thus, connectome-based models capable of predicting the depression severity at the individual level can be clinically useful. Here, we applied a machine-learning approach to predict the severity of depression using resting-state networks derived from source-reconstructed Electroencephalography (EEG) signals. Using regression models and three independent EEG datasets (N = 328), we tested whether resting state functional connectivity could predict individual depression score. On the first dataset, results showed that individuals scores could be reasonably predicted (*r* = 0.6, *p* = 4 × 10^–18^) using intrinsic functional connectivity in the EEG alpha band (8–13 Hz). In particular, the brain regions which contributed the most to the predictive network belong to the default mode network. We further tested the predictive potential of the established model by conducting two external validations on (N1 = 53, N2 = 154). Results showed statistically significant correlations between the predicted and the measured depression scale scores (*r1* = 0.52, *r2* = 0.44, *p* < 0.001). These findings lay the foundation for developing a generalizable and scientifically interpretable EEG network-based markers that can ultimately support clinicians in a biologically-based characterization of MDD.

## Introduction

Major Depressive Disorder (MDD) is one of the most common psychiatric disorders, mainly characterized by anhedonia and disturbed mood affecting the patient's quality of life and psychological state and has implications for their social and economic conditions^1^. Due to its increasing prevalence, chronicity, recurrence and degraded quality of life, MDD is now considered as a public health problem^2,3^. Currently, there are no biological signature of MDD, most probably because of its heterogeneity, and therefore prognosis (including better treatment response) and even diagnostic can be, sometimes, challenging. Researchers in mental illness advocate for a more biologically based framework to diagnose and treat these disorders, including depression^4,5^. At the cerebral level, most recent advances have moved from localized cerebral area disruptions to more network-based measures of mental disorders^6^.

Emerging evidence across functional studies consistently points at disruptions of MDD brain networks both in resting state^7–19^ and during task-based connectivity^20–22^. Specifically, functional abnormalities in network topology^11,20,21^, modularity^10,22^, and efficiency^8,11,15,16,23^ have been detected between MDD and healthy controls. are predominantly observed in the default-mode network (DMN) regions, including the posterior cingulate^23,24^, hippocampus^7,8,23,25^, parahippocampal gyrus^7,21,23^, precuneus^23,26^ superior parietal lobule^18,23,26^, and the executive network (ECN) including dorsolateral prefrontal cortex^8,9,14,25^ and the anterior cingulate cortex^8–10,13,15–18,20,21,25^. Importantly, DMN abnormal connectivity patterns is thought to be related with the cognitive vulnerability^27,28^ and negative self-referential thoughts of MDD patients^29,30^.

While this previous work has enabled a step toward a better understanding of the underlying pathophysiology of MDD, their translational potential into clinical use is hampered by the cost-effectiveness and availability of MRI. Electroencephalography (EEG) combined with the use of easy-to-implement measures of the brain network have been used to both binary classify^31–33^ and to predict treatment outcomes^34,35^ in MDD.

However, binary classification does not answer the question of MDD heterogeneity nor testing the possible continuum between normal sadness and pathological major depression. Very recently, resting state global connectivity measures predict both depressive and anxiety symptoms^36–38^. However, machine-learning/multivariate based connectivity measures are of high dimension and therefore often hampered by overfitting biases^39^. Proper analyses require large sample size and independent sample to validate the trained algorithm, which has not been the case in the field of EEG and affective disorders.

In this paper, we aim to establish a model that predicts depression severity based on resting-state EEG cortical networks. To tackle the problem of generalizability, three independent large datasets (N = 328) were used. Based on the prior findings, we hypothesized that the key regions that might contribute to the predictive model comprised DMN and ECN regions, mainly the anterior cingulate cortex, the prefrontal cortex, and the parahippocampal regions. We also expected that individual differences in MDD would be predicted by characteristics of alpha band as many studies suggest its relation to the pathologic characteristics of MDD^40–42^. EEG signals collected from 121 subjects (76 healthy controls and 45 MDD patients) were used to derive predictive models for depression severity. Brain networks were reconstructed using EEG source connectivity method^43^. Then, the Connectome-based Predictive Modeling (CPM)^44^ was applied in order to establish the relationship between functional brain networks and depression severity in support vector regression model. The external validation was performed using two other independent EEG datasets, where the first includes 24 MDD and 29 healthy controls, and the second (N = 154) -with no MDD patients- shows an ultimate perspective of predicting a possible continuum between normal sadness and pathological major depression.

## Materials and methods

**Figure 1:**
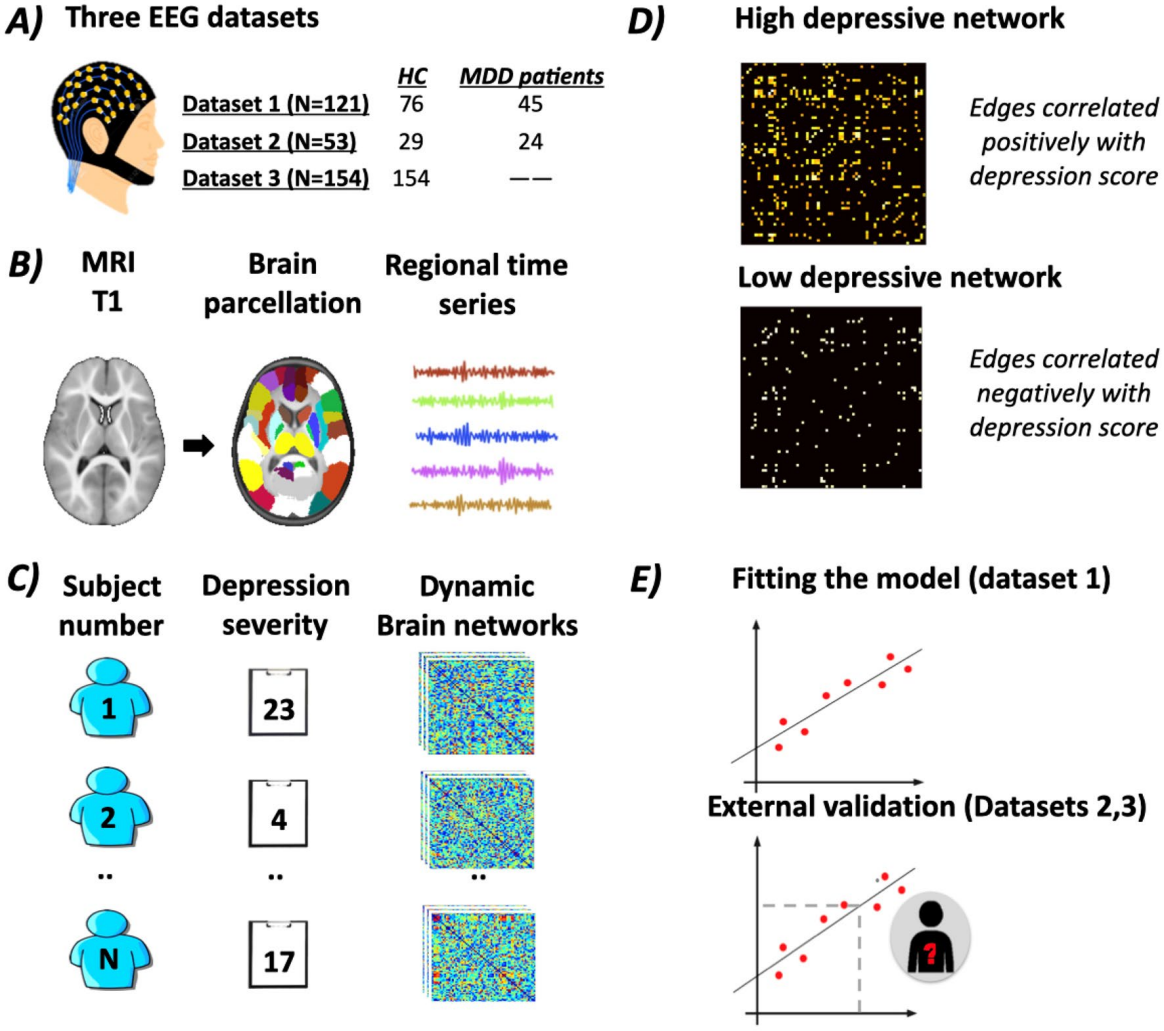
The full pipeline of our study. (**A**) EEG signals were acquired from three datasets: (1) N = 121 subjects (76 healthy controls and 45 MDD patients), (2) N = 53 subjects (29 healthy controls and 24 MDD patients) (3) N = 154 healthy subjects. (**B**) A template Magnetic Resonance Imaging (MRI) was segmented into 68 regions of interest (ROIs) by the means of Desikan Killiany atlas. Then, the regional time series of each subject were reconstructed using the weighted minimum norm estimate inverse solution (WMNE). (**C**) The inputs of the connectome-based predictive modeling (CPM) are the connectivity matrices and the depression score of each subject (BDI -i.e. dataset1-, Hamilton -i.e. dataset2, dataset 3-). The brain networks of each subject were obtained by computing the phase locking value between the regional time series. (b) The training step: across all subjects, each edge is correlated to the BDI score. Then, the algorithm selects the most important edges using significance testing (p < 0.01). Two matrices are thus resulted: the first corresponds to the network positively correlated with BDI and the second represents the network negatively correlated with BDI. (**E**) SVR predictive model is built to find the relationship between BDI and the sum of edge weights of the matrices obtained in the previous step. This model is then applied to predict the depression severity of subjects from the second and third dataset.

The full pipeline of this study is summarized in Fig. 1.

### First dataset

#### Participants

Forty-five MDD patients and seventy-six healthy controls have participated in the current study. The EEG database is publicly available at http://bit.ly/2rzY6ZY and was used in a recent previous study^45^. The study was approved by the ethical committee of Arizona University and experiments were in accordance with relevant ethical guidelines. Prior to acquisition, all participants provided written informed consent. Participants were recruited from introductory psychology classes based on mass survey scores of the Beck Depression Inventory (BDI). Recruitment criteria included: (1) age 18–25, (2) no history of head trauma or seizures, and (3) no current psychoactive medication use. Control participants (N = 76) have stable low BDI (< 7) between mass survey and preliminary assessment, no self-reported history of MDD, and no self-reported symptoms indicating the possibility of an Axis 1 disorder as indicated by computerized self-report completion of the Electronic Mini International Neuropsychological Interview (eMINI: Medical Outcome Systems, Jacksonville, FL, USA). Depressed participants have a stable high BDI (> 13). Participant demographics are reported in Table 1.

**Table 1:** Demographics of the 121 participants.

	HC	MDD	p-value
Cases (N)	76	45	NaN
Gender (M/F)	36/40	12/33	0.02
Age (years)	18.9 ± 0.6	19 ± 0.55	0.15
**Symptom scores**			
BDI	1.6 ± 0.75	22 ± 2.7	1E−20
BDI_Anh	0.16 ± 0.2	4 ± 0.8	1E−22
BDI_Mel	0.81 ± 0.4	6 ± 1	1E−17
TAI	30.87 ± 2.8	56 ± 4.6	1E−20

*BDI_Anh* anhedonia subscale of BDI, *BDI_Mel* Melancholia subscale of BDI, *TAI* trait anxiety inventory.

#### EEG acquisition and pre-processing

Participants were asked to stay relaxed for five minutes while EEG signals were recorded. Signals were acquired using 64 Ag/AgCl EEG electrodes (Synamps system) positioned according to the standard 10–20 system montage, two electro-oculogram electrodes (EOG) for horizontal and vertical movements. Signals were sampled at 500 Hz, bandpass filtered between 0.5 and 100 Hz. All electrodes’ impedances were kept below 10 kΩ.

Because of contamination of the EEG signals by various types of artifacts, EEG (pre)processing was applied following the same steps proposed in several previous studies dealing with EEG resting-state data^46–48^. These steps are summarized as follow: (1) identification of bad channels providing signals that are either completely flat or contaminated by movement artifacts. This was performed by visual inspection, complemented by the power spectral density, (2) interpolation of identified bad channels in Brainstorm by using neighboring electrodes within a 5-cm radius^49^. (3) segmentation into 40-s length epochs and only epochs with voltage fluctuations between + 100 μV and − 100 μV were retained. For each participant, four artifact-free epochs of 40-s lengths were selected. This epoch length guarantees a good compromise between the needed temporal resolution and the results reproducibility as demonstrated in^48^. Due to poor signal quality, EEGs from three HC subjects were excluded from the study.

### Second dataset

#### Participants

A total of 24 subjects (30.88 ± 10.37 years, female = 11) diagnosed with major depressive disorder, and 29 healthy controls (31.45 ± 9.15 years, female = 9) were recruited for the study. Patients with MDD were recruited from Lanzhou University Second Hospital, Gansu, China, diagnosed and recommended by professional psychiatrists while healthy subjects were recruited by posters. The study was approved by the Ethics Committee of the Second Affiliated Hospital of Lanzhou University. All procedures performed in the study were in accordance with the ethical guideline of the national research committee of Lanzhou University. Written informed consent was obtained from all subjects before the experiment began. All MDD patients received a structured MiniInternational Neuropsychiatric Interview (MINI) that meets the diagnostic criteria for major depression of Diagnostic and Statistical Manual of Mental Disorders (DSM) based on the DSM. The dataset is publicly available in. Depression symptoms were identified with Hamilton Depression Scale (HDRS).

#### EEG acquisition and pre-processing

During experiment, participants were required to relax, stay awake and try avoiding movements while 5 min eye-closed resting state EEG were recorded in a sound-proof room. 128-channel HydroCel Geodesic Sensor Net (Electrical Geodesics Inc., Oregon Eugene, USA) was used and electrode signals were referenced to Cz. During acquisition, each electrode kept an impedance below 50 kΩ. EEG preprocessing was performed in the same way dealt with the first dataset, leading to four artifact-free epochs of 40-s lengths for each participant.

### Third dataset

#### Participants

154 young healthy participants (N = 154, 25.1 ± 3.1 years, range 20–35 years, 45 female) were provided by the Mind-Brain-Body dataset^50^ publicly available in http://fcon_1000.projects.nitrc.org/indi/retro/MPI_LEMON.html. The study was carried out in accordance with the Declaration of Helsinki and the study protocol was approved by the ethics committee at the medical faculty of the University of Leipzig (reference number 154/13-ff). All included participants provided written informed consent before any data acquisition. The dataset originally included also 75 elderly subjects who were removed from the analysis to avoid the impact of age on the external validity of the model. Depression symptoms were identified with Hamilton Depression Scale^51^.

#### EEG acquisition and pre-processing

Participants were instructed to be awake with their eyes open and fixate on a low-contrast fixation cross on grey background. For each subject, 16-min resting state EEG was recorded with a ‘BrainAmp plus’ amplifier EEG using both 62-channel (61 scalp electrodes plus 1 electrode recording the VEOG below the right eye) and active ActiCAP electrodes (Brain Products GmbH, Gilching, Germany) positioned according to the international standard 10–20 extended localization system. Electrodes were referenced to FCz, the ground was located at the sternum and electrode impedance was kept below 5 KΩ. EEG were bandpass filtered between 0.015 Hz and 1 kHz and sampled at 2500 Hz.

Data were provided pre-processed, after passing through a pipeline that removed artefactual segments, identified faulty recording channels, and regressed out artefacts which appear as independent components in an Independent Component Analysis (ICA) decomposition with clear artefactual temporal signatures (such as eye blinks or cardiac interference).

### Brain networks construction

Brain networks were reconstructed using the “EEG source connectivity” method ^43^ following two main steps: (i) estimate the cortical sources and reconstruct their temporal dynamics by solving the inverse problem, and (ii) measure the functional connectivity between the reconstructed regional time-series.

Brain networks were reconstructed using the “EEG source connectivity” method^52^ using two main steps: (i) estimate the cortical sources and reconstruct their temporal dynamics using the inverse solution, and (ii) measuring the functional connectivity between the reconstructed regional time-series.

In brief, after co-registering EEGs and MRI template (ICBM152), a realistic head model was built using the OpenMEEG tool^53^. Then, the cortical surface was parcellated into 68 regions of interest by the means of Desikan-Killiany atlas^54^. As an inverse solution, the weighted minimum norm estimate (wMNE) algorithm was used to estimate the regional time series^55^. Afterwards, we filtered the reconstructed regional time series in the EEG frequency bands (delta: 1–4 Hz; theta: 4–8 Hz; alpha: 8–13 Hz; beta: 13–30 Hz and gamma: 30–45 Hz). In order to finally reconstruct the functional network, the Phase Locking Value (PLV) was assessed between the regional time series. This process applied yields an undirected and weighted connectivity matrix for each subject. We used the wMNE/PLV combination to reconstruct the dynamic networks, as it is widely used in the context of EEG source-space networks at rest^48,56^ and it is supported by several model-based and real data-based comparative studies^57^.

### Connectome predictive modeling

In this study, we are more interested in quantifying MDD heterogeneity in terms of brain connectivity rather than classifying binary groups. The binary classification will be very limited to deal with the MDD heterogeneity or testing the possible continuum between normal sadness and pathological major depression. Thus, we used the BDI scores, that quantify MDD severity, to construct the predictive networks of depression using the first dataset. This was performed using the connectome-based predictive modeling (CPM). Using the established model, external validation will be conducted using datasets 2 and 3. The CPM^44^ is a recently developed method for identifying and modeling a brain network associated with a variable of interest, the depression severity in our case. CPM was previously employed in a number of studies to predict network alteration in several brain disorders such as anxiety related illnesses^58^and sleep disorders^59^ as well in some other conditions such as personality traits and creativity^60–62^.

### Data preparation

Before establishing the predictive model, we prepared the inputs required for the prediction:

1- Preparation of the resting state functional connectivity matrices: For each subject, the connectivity matrices were averaged across time samples and epochs in order to obtain one representative matrix.

2- Preparation of the depression severity scores: Due to the difference in the measure used to assess the depression severity across datasets and their range of values (BDI scores (0–63) in Dataset 1 and HDRS in Datasets 2 and 3 (0–52)), it is recommended to use standardized measures in order to improve the interpretability of the results^63,64^. More specifically, the standardized BDI (BDI_s_) scores of dataset 1 were calculated by computing the difference between BDI and the distribution-mean divided by the standard deviation (Z-transformation). To ensure that the same scale was used across datasets, Z-transformation of the Hamilton scores corresponding to Datasets 2 and 3 was performed using the equivalent population-mean and standard deviation calculated on Dataset 1, yielding to standardized Hamilton scores (HDRS_s_). More precisely, we have first calculated the mean and the standard deviation of the BDI scores of the training set. Then, we have computed the equivalent mean and standard deviation following the so-called rule of three mathematical rule based on the HDRS range (0–52). Hence, the mean and the variance used in the Z-transformation of HDRS scores are obtained such that:

$$\mathrm{Equivalent}\_\mathrm{metric}\_\mathrm{HDRS}=\mathrm{metric}\_\mathrm{BDI}\times \frac{52}{63}$$

where metric is either the mean or the standard deviation, Equivalent_metric_HDRS is the equivalent value of the considered metric used in the normalization of Hamilton scores, metric_BDI is the value of the considered metric as calculated based on BDI distribution. Finally, HDRS_s_ was calculating by subtracting the equivalent_mean_HDRS and dividing by the equivalent_standard deviation_HDRS.

### Internal validation: prediction analysis using cross-validation

We established the predictive model using the first EEG dataset. To quantitatively assess the ability of the model in predicting novel samples, an internal validation using dataset 1 was performed. Thus, a fivefold cross validation was used to evaluate the prediction performance. Particularly, it was reported that K-fold cross-validation procedure provides an accurate model evaluation compared to holdout method and leave-one-out procedure^65^. Here, fivefold cross-validation method was performed by splitting the 121 subjects into 5 non-overlapping subsets of data (also known as folds). The assignment of subsets as training and testing data is repeated 5 times, where at each time, the subjects of 4 subsets are assigned as training data, with the remaining subset reserved as testing data. The training procedure includes the following steps:

1. *Edge-standardization* The weight of each edge was standardized (mean-centered) across the training set. Specially, we first computed the mean and standard deviation on each edge across subjects in the training set. Then, for each subject, we performed a Z-transformation on each edge by calculating the difference between its weight and the mean divided by the standard deviation. This step was performed in many previous CPM studies^61,62,66^).
2. *Identification of the predictive edges* Each edge in the connectivity matrix was correlated with the BDI_s_ scores for the training set using Pearson’s correlation. To calculate this correlation, the Z-transformed weight of the edge was used. Afterwards, only edges that show significant correlations with BDI_s_ (*p*_*FDR*_ < 0.01) were retained. In particular, the false discovery rate (FDR) approach was used to account for the multiple comparison problem. The correlation values were separated into a positive tail (i.e. edges correlated positively with BDIs scores) and a negative tail (i.e. edges correlated negatively with BDIs scores). Therefore, this step will result in the reconstruction of two networks: high depressive network (including connections in the positive tail) and low depressive network (including connections in the positive tail).
3. *Computation of the edge strength* The edge strengths were calculated in both positive and negative tails in order to correlate it with BDIs, as proposed by^44^. By combining both depressive networks, we also calculated the summed index. This latter is obtained by summing the Z-scores of all connections of the positive and subtracting those obtained from the negative tails.
4. *Construction of Support Vector Regression model (SVR)* SVR was employed to respectively relate positive and negative edge strengths to BDIs scores^67^.

Once the trained model is established using the training subset, the model is tested using the training fold. The testing procedure includes the following steps:

1. *Edge standardization* We have first standardized the edges of the depressive networks using the same parameters (i.e. mean and standard deviation) acquired during the training procedure.
2. *Testing the model* The trained SVR model was used to predict the testing participant's BDI scores.
3. *Assessing the predictive performance of the model* To evaluate the results of the prediction, we used several metrics: mean absolute error (MAE) which measures the difference between the predicted and the observed score, Pearson’s correlation (R) that measures the correlation between the predicted and the observed score, and the coefficient of determination, (R-squared) that reports the proportion of the variation in the dependent variable (depressive score) that is predictable from the independent variable (edge strength).

The fivefold cross validation was repeated for 100 iterations, and the resulted performance presents the average performance across all iterations and folds.

Finally, in order to check if the obtained correlation is significantly better than expected by chance, we performed a permutation test by permuting the BDI scores of all participants for 1000 times. In each permutation, we shuffled the BDIs scores in a random way and then we repeated the cross-validation procedure explained above including all the steps of the training and testing procedures. An averaged prediction error quantified by the mean absolute error (MAE) value across folds was obtained in each permutation. This yields in a null distribution of 1000 correlation values. Then, p-value was estimated after dividing the number of times the shuffled value was greater than the true MAE value by 1000 (the number of iterations). Furthermore, the prediction results were validated using a different cross‐validation scheme using K = 10, to see the consistency of results.

### The predictive networks

Due to the difference in the predictive networks obtained across iterations during the internal validation, the final generalized positive and negative predictive edges were defined as those that persist in all iterations. The derived common positive and negative networks will thus be used as the predictive networks for the external validation. The same procedure was applied in the previous connectome-based predictive modeling studies^58,66^.

### External validation

The derived positive and negative predictive networks obtained using the first dataset were used as predictive networks for the second and third independent dataset. Before computing the edge strength within the high and low depressive networks, edge z-transformation was conducted using the standardization parameters obtained on Dataset 1. Then, the SVR trained models using the predictive edge strengths were used for testing. The predictive performance was assessed by calculating the mean absolute error (MAE), Pearson’s correlation (R), the coefficient of determination (R-squared)^63,65^ and bootstrapping *p*-value.

### Confounder analysis

To explore potential confounding variables, we correlated age with the dependent variable (i.e. the depression severity) as well as with the independent variables that are the predictive selected features (i.e. the significant edges) using Pearson’s correlation. Similarly, Point-Biserial Correlation was used to correlate gender with the depression severity and the predictive edges. To avoid potential confounder effects of correlated variables, partial Pearson correlation analysis were conducted with the considered variable in edge selection as suggested by^58^.

## Results

### Demographics data

In dataset 1, the mean BDI is 9.5207 with a standard deviation of 10.5064. These values were used to obtain BDI_s_ that were the target variables in the model training. The equivalent values of the same parameters based on the Hamilton range were used to calculate the standardized Hamilton scores (HDRS_s_) that represent the target variables in external validation. BDI_s_ range from − 0.9 to 1.94 with a robust range (range between the 5th and 95th percentiles) of 1.73 (arbitrary units). HDRS_s_ in dataset 2 range from − 0.9062 to 4.5245 with an interquartile of 3.707, while HDRS_s_ in dataset 3 range from − 0.9062 to 0.7082 with an interquartile of 0.4613.

For dataset 1, no statistical difference in age (*p* = 0.15) was depicted between MDD and HC. Results show the existence of a statistical difference in gender (*p* = 0.02) as computed using chi-square test. MDD patients and HC differ in all the recorded psychiatric scores (*p* < 0.0001) as computed using the Wilcoxon ranksum test. For dataset 2, there were no significant between-group differences in age (*p* = 0.832), or gender (*p* = 0.269). Statistical differences in the self-reported PHQ-9, and the Generalized Anxiety Disorder-7 (GAD7), the Hamilton depressive scores were obtained (*p* < 0.001).

### Confounder control

Results showed that age was not significantly associated with BDIs nor the weights of any predictive edge derived in the training dataset (*p* > 0.2). Ultimately, age was not considered to be a confounding variable in our predictive model. While gender was not significantly correlated with BDI_s_ (*p* > 0.05), its effect size is found to be considerable (*r* = 0.15, *p* = 0.09). Thus, gender was considered as a confounding variable in the model estimation.

### Predictive networks

**Figure 2:**
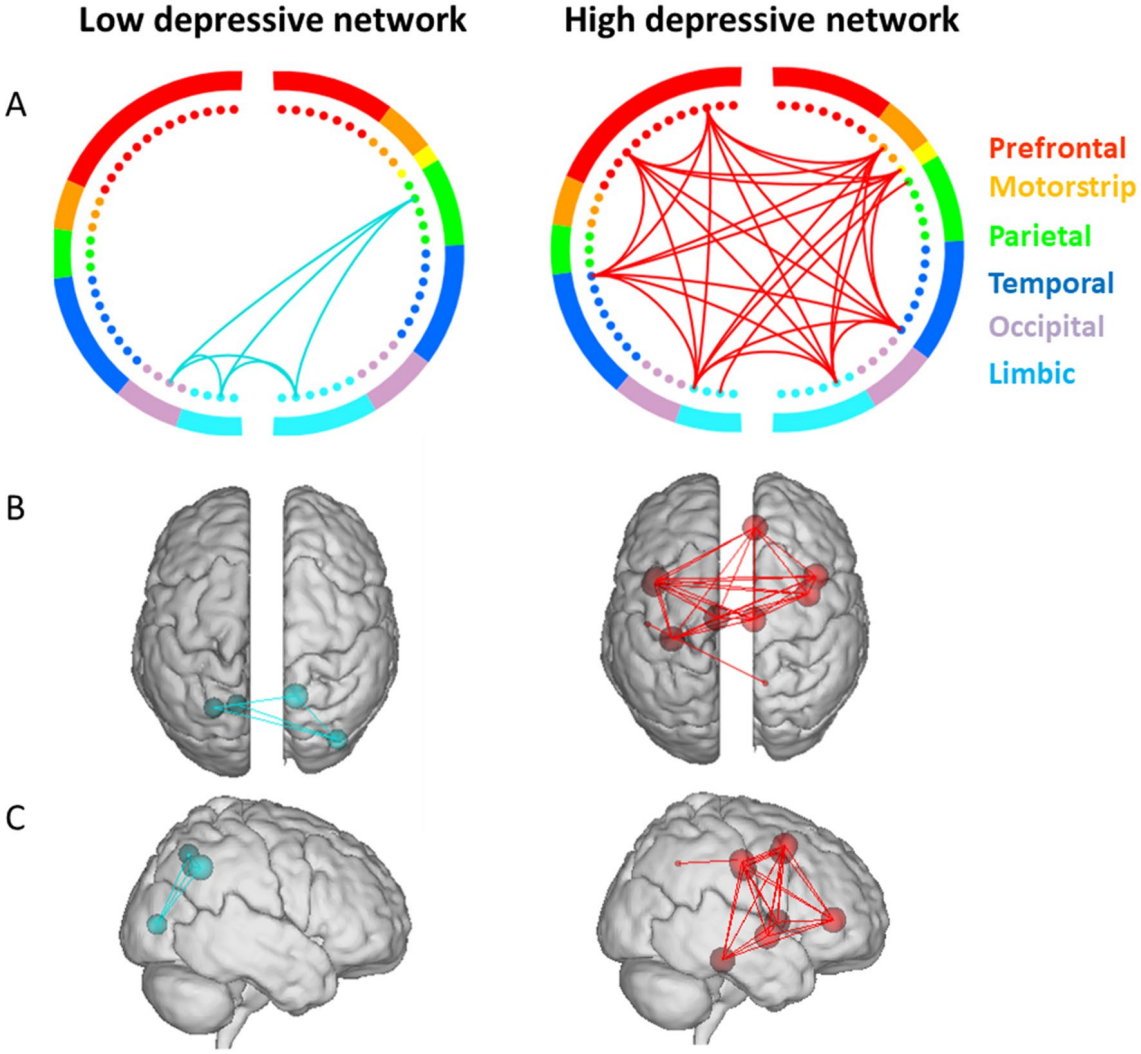
Depictions of the high- and low-depressive networks. Circle plots (**A**), glass brains—top view (**B**), glass brain- right view were obtained by keeping the significant edges (p < 0.01, FDR corrected). Colors within the circle plots correspond to lobes of the brain.

As mentioned in the materials section, the predictive networks were derived after applying the cross-validation procedure on dataset 1. Two main networks result: (1) The high depressive network which denotes the network in which edges show positive significant correlations with BDI_s_ scores, and (2) the low depressive network which denotes the network in which edges show negative significant correlations with BDI scores. Gender was considered as a covariate in the model estimation. No significant networks were detected in delta, theta, beta and gamma bands. In alpha band, the numbers of edges that contributed to the prediction ranged from 75 to 189 across all iterations. Notably, 38 of these edges (33 high depressive edges, 5 low depressive edges) appeared in every iteration and were defined as the depressive edges. The high depressive network exhibited dense functional connections in predominantly prefrontal, insula and limbic lobes (Fig. 2). More specifically, the implicated brain regions are: the caudal middle frontal gyrus (cMFG), insula (INS), parahippocampal (paraH), posterior cingulate (PCC), and rostral anterior cingulate (rACC). The low-depressive network showed edges within occipital, parietal and limbic lobes. Nodes are the lateral occipital gyrus (LOG), the superior parietal lobule (SPL) and the precunues (PCUN). Notably, the implicated regions in the predictive networks belong mainly to the default mode network (paraH, PCUN, PCC, SPL).

### Internal validation using dataset 1: cross validation

**Figure 3:**
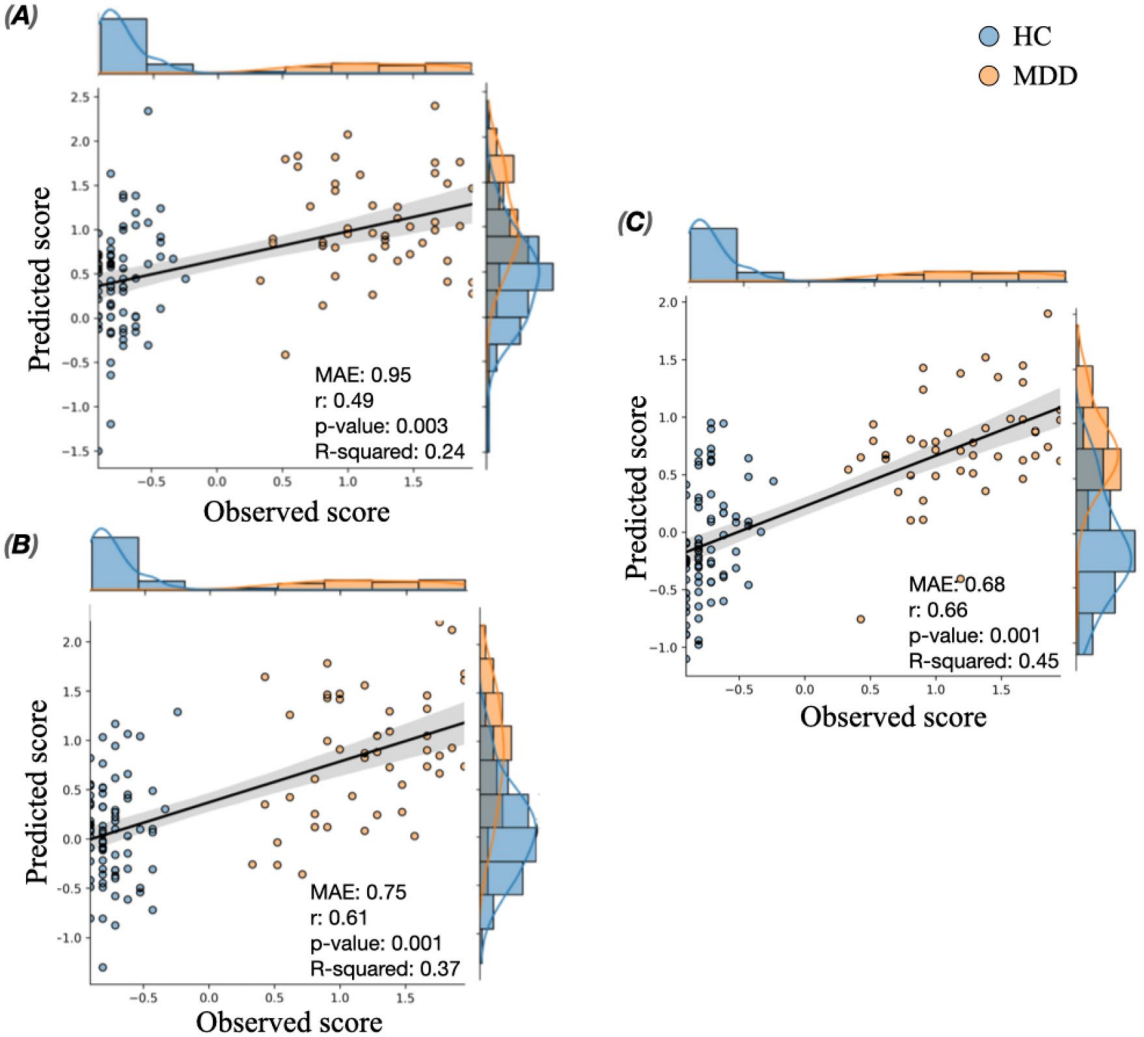
A typical example (chosen from a random iteration) showing the relationship between the observed and the predicted scores using (**A**) low depressive edges, (**B**) high depressive edges, (**C**) combined depressive edges.

As the predictive performance differs across folds/iterations, we reported here the average performance metrics along with a measure of variability across folds/iterations (i.e. the standard deviation). Results showed that the model designed based on the predictive edges of the low depressive network (showing negative correlation with BDIs) were able to predict BDIs scores in novel individuals (*r* = *0*.61 ± 0.08; *MAE* = *0.68* ± 0.09, R-squared = 0.48 ± 0.08, *p* = 0.001). Figure S3 shows the corresponding histogram of the null MAE distribution. According the high depressive network, the predictions obtained from of the model achieved *r* = *0*.42 ± 0.09, *MAE* = 0.94 ± 0.06, R-squared = 0.23 ± 0.05 with *p* = 0.003 (see Fig. S2). Figure 3A,B illustrate an example of the internal validation obtained for the low and high depressive networks, respectively. The illustrated examples are picked up from a single representative iteration chosen randomly on condition that the corresponding MAE value resides in the interquartile of the MAE distribution across iterations.

We also evaluated the predictions of the model that combined both high and low depressive edges. Our findings reveal a positive significant correlation of *r* = 0.59 ± 0.1, between the observed and the predicted BDI_s_, with R-squared = 0.46 ± 0.07, MAE = 0.75 ± 0.091 with *p* = 0.001 (Fig. S1). An example is shown in Fig. 3C.

Results obtained using tenfold cross validation show consistent results (see Table 2).

**Table 2:** Results of the internal validation using fivefold and tenfold cross validations.

	Fivefold cross validation		Tenfold cross validation	
	High depressive edges	Low depressive edges	High depressive edges	Low depressive edges
r	*0*.42 ± 0.09	*0*.61 ± 0.08	*0*.47 ± 0.13	*0*.65 ± 0.11
MAE	0.95 ± 0.06	*0.48* ± 0.09	0.91 ± 0.1	*0*.42 ± 0.09
Permutation-based p	0.009 ± 0.006	0.005 ± 0.003	0.006 ± 0.009	0.004 ± 0.006
R-squared	0.23 ± 0.05	0.48 ± 0.08	0.21 ± 0.05	0.43 ± 0.05

r denotes the Pearson’s correlation between observed and predicted scores. MAE denotes the mean absolute error of the prediction. R-squared presents the coefficient of determination.

### External validation on datasets 2 and 3

**Figure 4:**
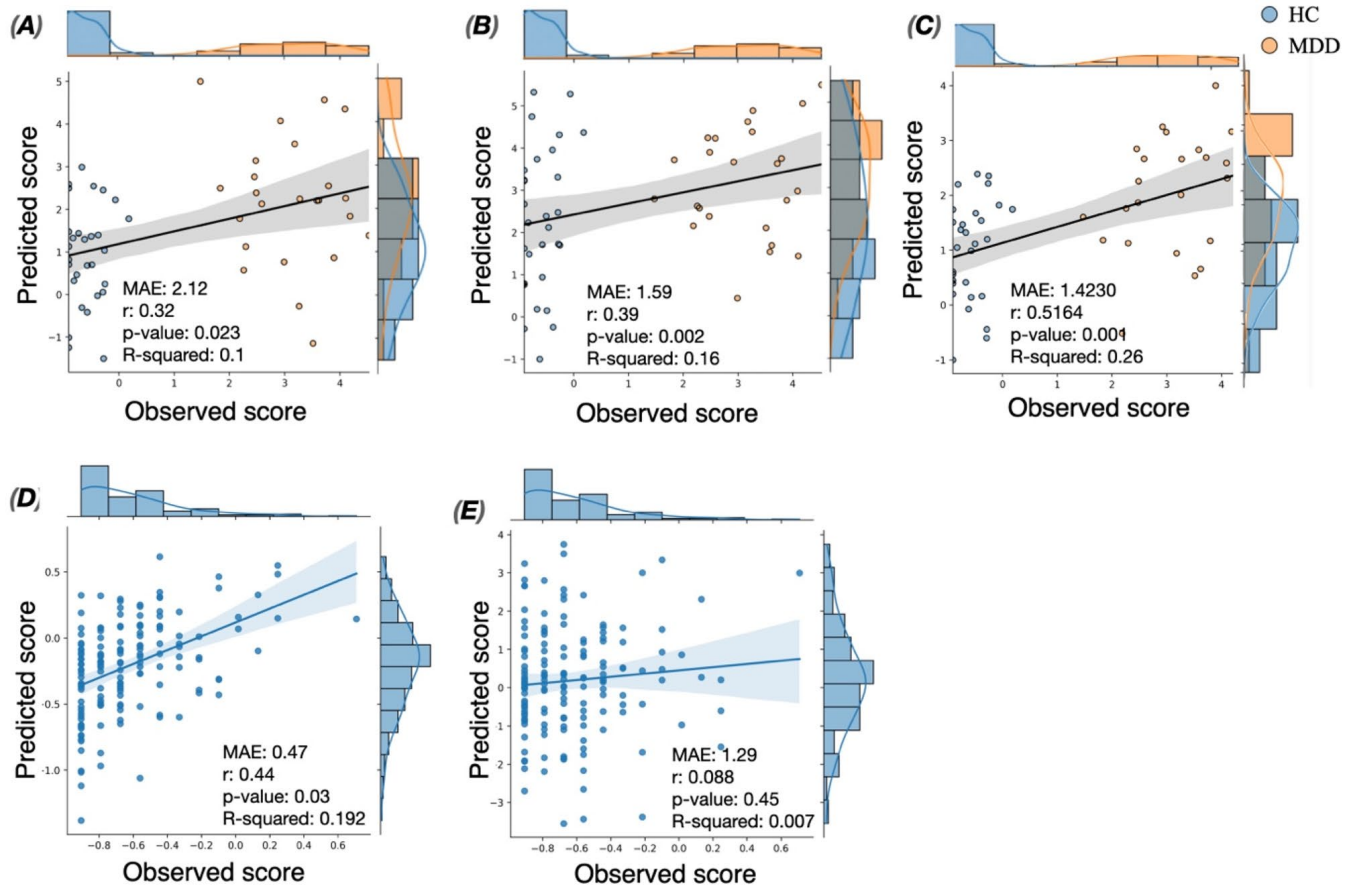
(**A**) Correlation between the predicted and observed Hamilton scaling score of the second dataset using high-depressive network, (**B**) the prediction results of the second dataset using low-depressive network, (**C**) the prediction results of the second dataset using both high and low depressive network. (**D**) The prediction results of the third dataset using high depressive network. (**E**) The prediction results of the third dataset using low depressive network.

Using the model developed based on the high depressive edges, the performance of the prediction achieved (r = 0.3965, MAE = 1.5984, R-squared = 0.1572, *p* = 0.002 (Fig. S4, Fig. 4A). In addition, statistically significant prediction was obtained using the low depressive connections (r = 0.3198, MAE = 2.1268, R-squared = 0.1023, *p* = 0.02 (Fig. S5, Fig. 4B). Using both predictive networks, the prediction achieved r = 0.5164, MAE = 1.4230, R-squared = 0.2666 with *p* = 0.001 (Fig. S6, Fig. 4C).

Our findings reveal that the SVR model was able to predict the depression scores for the high-depressive network (r = 0.4391; M*AE* = *0.4700*, R-squared = 0.1928, *p* = 0.03; Fig. 4D, Fig. S7), but not for the low-depressive network (*r* = *0.0881, MAE* = *1.2980*, R-squared = 0.0078, *p* = *0.45;* Fig. S8, Fig. 4E).

## Discussion

The objective of this study is to establish a connectome-based model capable of predicting depression score at the individual level. Using three independent resting state EEG datasets, our findings proved that depression could be predicted using intrinsic functional connectivity. More specifically, the majority of the nodes that contributed the most to the predictive network belong to the DMN obtained in the alpha frequency band. Interestingly, generalizability of the model to predict novel and independent samples was demonstrated across different datasets as well as across different psychometric tests (BDI, HDRS).

The model predicts a considerable amount of the variance in individual depression symptom severity (48% with internal validation and 37%–19% with external validation). The obtained MAE obtained in internal and external validations suggest a good generalizability of the results. Results are discussed hereafter.

### Brain regions implicated in predictive network

Alterations in the paraH connectivity were previously reported^7,21,23^. As this region plays a major role in memory retrieval, it is supposed that such alteration is associated with the recall or the imagination of negative memories and events in MDD patients^68^. Consistent with our findings, previous studies have reported higher activation of paraH in depressed subjects compared to healthy group during rumination (moments)^69^.

In addition, increased blood flow in left and right PCC regions were associated with MDD^70^. Using EEG source connectivity combined with graph theory, a recent EEG study shows that MDD brain network is characterized by increased efficiency in PCC^23^. Importantly, PCC is a key region in the limbic system and the DMN, and has a major role in regulating emotions and motivational thoughts^71^. Similarly, ACC which has been associated with MDD in multiple studies^8–10,13,15–18,20,21,25^, has been most frequently linked to the experience of pain, and specially in monitoring painful social situations such as exclusion or rejection^72^. This may explain the implication of PCC and ACC in the high depressive network. Furthermore, the prefrontal and limbic cortical regions are directly modulated by the subcortical structures that are involved in the negative emotional circuits. For instance, the canonical amygdala, hippocampal structures, and the lateral habenula (LHb) has been found to represent key brain regions in the pathophysiology of depression^73^. Clinical and preclinical evidence implicates hyperexcitability of the lateral habenula (LHb) in the development of psychiatric disorders including^74^.Hyperactive LHb strongly inhibit the dopaminergic motivational, cognitive and reward system^75^ and produces depressive- and anxiety-like behaviors, anhedonia and aversion. Dopaminergic and anxiety circuits strongly modulate the limbic cortex, including the orbitofrontal cortex and the anterior cingulate cortex. The medial prefrontal cortex in rodent, the homologue of the anterior cingulate cortex of human are strongly involved in the expression of anxiety and are involved in MDD^76^. Because these internal emotional circuits strongly modulate the prefrontal, insula and limbic lobes, it is not surprising that in the high depressive network we found a dense functional connection between these areas.

According to the low depressive network, the regions implicated are SPL, LOG and PCUN. In fact, SPL, LOG and PCUN connectivity were found to be different between MDD patients and healthy controls in many studies^18,23,26^. SPL is principally involved in attention and visuomotor integration^77^, whilst LOG plays a major role in the visual perception. In addition, visuospatial processing, reflections upon self, and aspects of consciousness are associated with PCUN. This may explain the implication of these regions in low depressive subjects where attentional and visual performances are more efficient.

### EEG frequency bands in depression

In this study, differences between MDD and healthy groups were only depicted in alpha band, and no significant results were obtained in other EEG frequency bands. Interestingly, using the same frequency band, the model has demonstrated its performance in predicting novel and independent samples. This finding is in line with a number of resting state EEG studies that continuously report MDD alterations in alpha frequency patterns. Particularly, alpha-asymmetry was suggested to be a potential marker for depression^26,40,78,79^ whereas alterations in other frequency band such as delta, beta and gamma power were inconsistent^40^. More importantly, a connectivity-based EEG analysis suggests an increase in alpha-band connectivity between the anterior cingulate cortex and both the prefrontal cortex^80^. It has also been proposed that reduced alpha desynchronization in a network involving bilateral frontal and right-lateralized parietal regions may provide a specific measure of deficits in approach-related motivation in depression^81^. Using graph theoretical methods, another study shows that disrupted global and local network indices in MDD patients were revealed in alpha band^11^. In addition, features derived from alpha band have been revealed to be highly discriminating when distinguishing between MDD patients and healthy controls using binary classifiers^32,34,37,82^. These observations may be explained by the fact that alpha rhythm is thought to functionally inhibit cortical responses to unattended components of sensory input^83^ and monitor important roles in both cognitive, social and emotion processing^84,85^ that are closely altered in MDD. In contrast, a recent study show that resting state connectivity disruptions emerged mainly in beta frequency band (12.5–21 Hz), supporting the hypothesis that beta-band synchronization corresponds to a cognitive idling rhythm present in MDD^21^. In addition, a large study including 1344 participants showed increases in source EEG theta power across frontal regions of the brain^86^. This inconsistency in the results might be due to the difference in the subjects/patients characteristics as well the difference in methodological issues such as number of subjects, data pre-processing and data analysis (power spectra vs. connectivity for instance). Moreover, neural connectivity networks likely involve coordinated synchronizations across frequencies. Thus, extending the analysis to establish a model based on cross-frequency couplings for instance may be of interest.

### Limitations and methodological considerations

First, the model was established using the first dataset composed of two groups (healthy and MDD) where the severity of depressive symptoms was assessed using BDI score. Although the BDI is a self-report questionnaire, as compared to the HDRS, was reported that BDI revealed high reliability and good correlation with measures of depression and anxiety^87^. However, like any self-report measure, BDI suffers from intrinsic limitations. Specifically, the score can be exaggerated or minimized based on the clinical environment^88^, fatigue^89^, and compromised cognitive functioning^87^.

Second, we are aware that subcortical regions are not easily accessible using scalp EEG, namely due to anatomical considerations. Unlike the layered cortex, a subcortical region would not have the necessary organization of pyramidal cells to give rise to localizable scalp-recorded EEG. Conversely, exploring subcortical regions may contribute to the modeling of depressive networks as many studies linked MDD with disruptions in amygdala^7,18,90^, a region centrally implicated in emotional and physiologic responses.

Third, a limitation of the current study is that one of the datasets included only healthy subjects and that some subjects experiencing mild depressive symptoms. We also believe that the correlation between observed and predicted HDRS scores needs to be validated in a larger sample (including MDD patients). In addition, longitudinal data (EEG data recorded from healthy subjects and MDD patients at several time points) is mandatory to validate and ultimately generalize the potential neuromarker.

Fourth, while the low-depressive network showed significant correlation between observed and predicted BDI scores in the Dataset 2, the external validation didn’t reveal significant correlations between observed and predicted HDRS scores in Dataset 3. This observation suggests that the high-depressive network showed consistent prediction of depression severity across both datasets while the low-depressive network is less relevant to the prediction performance for Dataset 3. It is worth noting, however, that the Dataset 2 provided two separated group of subjects (healthy and MDD patients), while Dataset 3 didn’t show a wide distribution of the HDRS score. More precisely, this latter mainly presents healthy subjects (with Hamilton scores spanning from 0 to 10) and some subjects experiencing mild depressive symptoms (Hamilton scores spanning from 10 to 14). This may explain the disability of the low-depressive network to discriminate between these subjects as it is expected to show similar low-network strength. Another explanatory possibility would be that normal sadness and pathological depression share the same continuum where major depression is defined based on the functional consequences of the symptoms (i.e. described as the pragmatic approach^91,92^. The continuum hypothesis between normal sadness and pathological major depression has also been supported at the symptom^93^ and the cerebral levels^94^ using data-driven latent analysis.

Additionally, we believe that a reliable interpretation of the predictive regression model is essential for clinical impact. One important issue that should be addressed is whether the significant correlation obtained between the predicted and the observed scores in Dataset 1 and 2 is biased by the two groups (HC and MDD) showing separated depression scores (see histograms in Figs. 3 and 4). To target this question, we have first assessed the predictive performance of the models not only using the correlation metric, but also using MAE and R-squared that are deemed to be more rigorous and sensitive to the scale of the prediction error. In addition, to quantify how much the prediction performances obtained are above/below chance, we employed a baseline regressor (also known as dummy regressor) that uses the average score of the training set as prediction. For the first dataset, results show that the established models (using high, low and both depressive networks as features) achieved MAE better than the error obtained by the dummy model, with no overlap between dummy MAE distributions (MAE = 1.18 ± 0.067) and MAE obtained by the model (MAE = 0.94 ± 0.06 using high depressive edges, MAE = 0.68 ± 0.09 using low depressive edges, MAE = 0.75 ± 0.091 when combining high and low depressive edges). For the second dataset, all but the model established using low depressive edges performed better than the dummy revealing MAE = 1.73. To better assess if the correlations obtained are biased by the bimodal distributions, we performed a group-wise evaluation separately for MDD and HC. The correlation results (mean ± std across folds) of Dataset 1 are reported in Table S1. For this dataset, results show that the group-wise correlations between the observed and predicted scores are considerable (> 0.35) when both high and low depressive edges are used as predictive features. However, the use of high depressive edges solely results in acceptable correlations for the HC group but not for the MDD group. In contrast, the low depressive edges revealed considerable correlations in MDD group but not in HC group. A possible explanation is that the high (low) depressive edges are more related to the HC (MDD) group as their weights are positively (negatively) correlated with BDI scores. This issue highlights the importance of combining both high and low depressive networks as predictive features, especially in the bimodal case, as each predictive network (high/low) better describes a particular group. For dataset 2, results revealed that the predictive performance of the model separately for MDD is weak using each predictive network solely (Table S2). As in Dataset 1, the combination of both predictive edges (i.e. high and low) underlies a better performance of the model for both HC and MDD groups. This observation may be related to the difference in the data distribution between dataset 1 (showing HC subjects remarkably more than MDD patients) and dataset 2.

We should also note that the prediction error obtained in Dataset 3 (MAE = 0.47) is not sufficiently small taking into account the min–max range (1.6) and the interquartile (0.46) of the dataset. However, the permuted based p-value shows that the predictive performance is significant (*p* < 0.05). In addition, the dummy mean model (returning the average of the training set as predicted score) resulted in MAE of 0.64, which suggests that the model performs better than expected by chance. On the other side, we are aware that the clinical needs require more robust predictions in terms of MAE, and R-squared.

Furthermore, internal validation was here assessed using a k-fold (fivefold and tenfold) cross validation procedure^63,65^. Although leave-one-out is the most popular cross validation method, it has been demonstrated that the k-fold cross validation provides more accurate prediction error. However, the choice of the number of folds (K) is critical and one should be prudent in K selection to ensure a good compromise between model bias and variance^95–97^. Referred to^65^, it is recommended to use 10 to 20% of data for the testing sample size. Thus, fivefold cross validation was used for the internal validation using the first dataset of 121 subjects so that 20% of data was used as testing sample in each iteration. Results of the internal validation using fivefold cross validation were also consistent with those obtained using a tenfold cross validation, suggesting that the main observations remain intact.

Previous studies showed that depression symptom severity strongly differ between elderly and young populations^98^. In the current study, we established the predictive model using resting-state functional connectivity acquired exclusively from young adults (< 40 years). To better ensure that age is not correlated with the depression severity scores, we controlled for its confounding effects. It is noteworthy to mention that other demographic information (such as education background, years and drugs) should be, when available, collected and controlled in future studies.

In conclusion, our findings demonstrate the feasibility of resting-state EEG whole-brain connectome in predicting individual differences in depression severity, suggesting that it might serve as direct and non-invasive neuromarker that can ultimately support clinicians in a biologically-based characterization of MDD, which eventually will improve MDD prognostic and therapeutic decision.

## Supplementary Information

Supplementary Information.

## References

[1] Simon SA, Nicolelis MAL (2011). Neurobiology of. Depression.

[2] Marcus, M., Yasamy, M. T., van Ommeren, M., & Chisholm, D. Depression, a global public health concern. *WHO Department of Mental Health and Substance Abuse* (2012).

[3] Cassanol, P., & Fava, M. Depression and public health: An overview. 10.1016/S0022-3999(02)00304-5 (2002). 10.1016/s0022-3999(02)00304-5 12377293

[4] Insel T (2010). Research domain criteria (RDoC): Toward a new classification framework for research on mental disorders. Am. J. Psychiatry.

[5] Schumann G (2014). Stratified medicine for mental disorders. Eur. Neuropsychopharmacol..

[6] Bullmore E, Sporns O (2009). Complex brain networks: Graph theoretical analysis of structural and functional systems. Nat. Rev. Neurosci.

[7] Cullen KR (2014). Abnormal amygdala resting-state functional connectivity in adolescent depression. JAMA Psychiat..

[8] Ye M, Yang T, Qing P, Lei X, Qiu J, Liu G (2015). Changes of functional brain networks in major depressive disorder: A graph theoretical analysis of resting-state fMRI. PLoS ONE.

[9] Sheline YI, Price JL, Yan Z, Mintun MA (2010). Resting-state functional MRI in depression unmasks increased connectivity between networks via the dorsal nexus. Proc. Natl. Acad. Sci. U.S.A..

[10] Yu Z (2020). Abnormal topology of brain functional networks in unipolar depression and bipolar disorder using optimal graph thresholding. Prog. Neuro-Psychopharmacol. Biol. Psychiatry.

[11] Shim M, Im CH, Kim YW, Lee SH (2018). Altered cortical functional network in major depressive disorder: A resting-state electroencephalogram study. NeuroImage Clin..

[12] Damborská A (2019). EEG resting-state large-scale brain network dynamics are related to depressive symptoms. Front. Psych..

[13] Greicius MD (2007). Resting-state functional connectivity in major depression: abnormally increased contributions from subgenual cingulate cortex and thalamus. Biol. Psychiat..

[14] Anand A, Li Y, Wang Y, Lowe MJ, Dzemidzic M (2009). Resting state corticolimbic connectivity abnormalities in unmedicated bipolar disorder and unipolar depression. Psychiatry Res. Neuroimaging.

[15] Dutta A, McKie S, Deakin JFW (2014). Resting state networks in major depressive disorder. Psychiatry Res. Neuroimaging..

[16] Hou Z (2016). Divergent topological architecture of the default mode network as a pretreatment predictor of early antidepressant response in major depressive disorder. Sci. Rep..

[17] Sheline YI, Price JL, Yan Z, Mintun MA (2010). Resting-state functional MRI in depression unmasks increased connectivity between networks via the dorsal nexus. Proc. Natl. Acad. Sci. U.S.A..

[18] Connolly CG (2013). Resting-state functional connectivity of subgenual anterior cingulate cortex in depressed adolescents. Biol. Psychiat..

[19] Zhou Y (2012). Early altered resting-state functional connectivity predicts the severity of post-traumatic stress disorder symptoms in acutely traumatized subjects. PLoS ONE.

[20] Wang X, Öngür D, Auerbach RP, Yao S (2016). Cognitive vulnerability to major depression: View from the intrinsic network and cross-network interactions. Harv. Rev. Psychiatry.

[21] Albert KM, Potter GG, Boyd BD, Kang H, Taylor WD (2019). Brain network functional connectivity and cognitive performance in major depressive disorder. J. Psychiatr. Res..

[22] Bi K (2018). Abnormal early dynamic individual patterns of functional networks in low gamma band for depression recognition. J. Affect. Disord..

[23] Whitton AE, Deccy S, Ironside ML, Kumar P, Beltzer M, Pizzagalli DA (2018). Electroencephalography source functional connectivity reveals abnormal high-frequency communication among large-scale functional networks in depression. Biol. Psychiatry Cognit. Neurosci. Neuroimaging.

[24] Wei M (2017). Abnormal dynamic community structure of the salience network in depression. J. Magn. Reson. Imaging.

[25] Chen T (2017). Anomalous single-subject based morphological cortical networks in drug-naive, first-episode major depressive disorder. Hum. Brain Mapp..

[26] Greicius MD (2007). Resting-state functional connectivity in major depression: Abnormally increased contributions from subgenual cingulate cortex and thalamus. Biol. Psychiat..

[27] Jiang X (2019). Connectome analysis of functional and structural hemispheric brain networks in major depressive disorder. Transl. Psychiatry.

[28] Kaiser RH, Andrews-Hanna JR, Wager TD, Pizzagalli DA (2015). Large-scale network dysfunction in major depressive disorder: A meta-analysis of resting-state functional connectivity. JAMA Psychiat..

[29] Broyd SJ, Demanuele C, Debener S, Helps SK, James CJ, Sonuga-Barke EJS (2009). Default-mode brain dysfunction in mental disorders: A systematic review. Neurosci. Biobehav. Rev..

[30] Gusnard DA, Akbudak E, Shulman GL, Raichle ME (2001). Medial prefrontal cortex and self-referential mental activity: Relation to a default mode of brain function. PNAS.

[31] Thoduparambil PP, Dominic A, Varghese SM (2020). EEG-based deep learning model for the automatic detection of clinical depression. Phys. Eng. Sci. Med..

[32] Uyulan C (2020). Major depressive disorder classification based on different convolutional neural network models: Deep learning approach. Clin. EEG Neurosci..

[33] Xie Y (2020). Anxiety and depression diagnosis method based on brain networks and convolutional neural networks. Annu. Int. Conf. IEEE Eng. Med. Biol. Soc..

[34] Rolle CE (2020). Cortical connectivity moderators of antidepressant vs placebo treatment response in major depressive disorder: Secondary analysis of a randomized clinical trial. JAMA Psychiat..

[35] Zhdanov A (2020). Use of machine learning for predicting escitalopram treatment outcome from electroencephalography recordings in adult patients with depression. JAMA Netw. Open.

[36] Mahato S, Goyal N, Ram D, Paul S (2020). Detection of depression and scaling of severity using six channel EEG data. J. Med. Syst..

[37] Mohammadi Y, Moradi MH (2020). Prediction of depression severity scores based on functional connectivity and complexity of the EEG signal. Clin. EEG Neurosci..

[38] Trambaiolli LR, Biazoli CE (2020). Resting-state global EEG connectivity predicts depression and anxiety severity. Annu. Int. Conf. IEEE Eng. Med. Biol. Soc..

[39] Jollans L (2019). Quantifying performance of machine learning methods for neuroimaging data. Neuroimage.

[40] Jaworska N, Blier P, Fusee W, Knott V (2012). Alpha power, alpha asymmetry and anterior cingulate cortex activity in depressed males and females. J. Psychiatr. Res..

[41] Knott V, Mahoney C, Kennedy S, Evans K (2001). EEG power, frequency, asymmetry and coherence in male depression. Psychiatry Res. Neuroimaging.

[42] Fingelkurts AA, Fingelkurts AA, Rytsälä H, Suominen K, Isometsä E, Kähkönen S (2007). Impaired functional connectivity at EEG alpha and theta frequency bands in major depression. Hum. Brain Mapp..

[43] Hassan M, Wendling F (2018). Electroencephalography source connectivity. IEEE Signal Process. Mag..

[44] Shen X (2017). Using connectome-based predictive modeling to predict individual behavior from brain connectivity. Nat. Protoc..

[45] Cavanagh JF, Bismark AW, Frank MJ, Allen JJB (2019). Multiple dissociations between comorbid depression and anxiety on reward and punishment processing: Evidence from computationally informed EEG. Comput. Psychiatry.

[46] Kabbara A, Eid H, Falou EL, Khalil M, Wendling F, Hassan M (2018). Reduced integration and improved segregation of functional brain networks in Alzheimer’s disease. J. Neural Eng..

[47] Rizkallah J, Benquet P, Kabbara A, Dufor O, Wendling F, Hassan M (2018). Dynamic reshaping of functional brain networks during visual object recognition. J. Neural Eng..

[48] Kabbara A, Falou WEL, Khalil M, Wendling F, Hassan M (2017). The dynamic functional core network of the human brain at rest. Sci. Rep..

[49] Tadel F, Baillet S, Mosher JC, Pantazis D, Leahy RM (2011). Brainstorm: A user-friendly application for MEG/EEG analysis. Comput. Intell. Neurosci..

[50] Babayan A (2019). Data descriptor: A mind-brain-body dataset of MRI, EEG, cognition, emotion, and peripheral physiology in young and old adults. Sci. Data.

[51] Hamilton M (1960). A rating scale for depression. J. Neurol. Neurosurg. Psychiatry..

[52] Hassan M, Wendling F (2018). Electroencephalography source connectivity: toward high time/space resolution brain networks. IEEE Signal Process. Mag..

[53] Gramfort A, Papadopoulo T, Olivi E, Clerc M (2010). OpenMEEG: Opensource software for quasistatic bioelectromagnetics. Biomed. Eng. Online.

[54] Desikan RS (2006). An automated labeling system for subdividing the human cerebral cortex on MRI scans into gyral based regions of interest. Neuroimage.

[55] Hamalainen MS, Ilmoniemi RJ (1994). Interpreting magnetic fields of the brain: Minimum norm estimates. Med. Biol. Eng. Compu..

[56] Mheich A (2021). HD-EEG for tracking sub-second brain dynamics during cognitive tasks. Sci. Data.

[57] Hassan M (2017). Identification of interictal epileptic networks from dense-EEG. Brain Topogr..

[58] Wang, Z., Goerlich, K. S., Ai, H., Aleman, A., Luo, Y., & Xu, P. Connectome-based predictive modeling of individual anxiety. *bioRxiv*, 2020.01.30.926980. 10.1101/2020.01.30.926980 (2020). 10.1093/cercor/bhaa407 33511990

[59] Giancardo, L., et al. Longitudinal connectome-based predictive modeling for REM sleep behavior disorder from structural brain connectivity. 10.1117/12.2293835 (2018).

[60] Beaty RE (2018). Robust prediction of individual creative ability from brain functional connectivity. Proc. Natl. Acad. Sci. U.S.A..

[61] Yoo K (2018). Connectome-based predictive modeling of attention: Comparing different functional connectivity features and prediction methods across datasets. Neuroimage.

[62] Feng C, Wang L, Li T, Xu P (2019). Connectome-based individualized prediction of loneliness. Social Cognit. Affect. Neurosci..

[63] Scheinost D (2019). Ten simple rules for predictive modeling of individual differences in neuroimaging. Neuroimage.

[64] Rosenberg MD (2016). A neuromarker of sustained attention from whole-brain functional connectivity. Nat. Neurosci..

[65] Poldrack RA, Huckins G, Varoquaux G (2020). Establishment of best practices for evidence for prediction: A review. JAMA Psychiat..

[66] Feng C (2018). Individualized prediction of trait narcissism from whole-brain resting-state functional connectivity. Hum. Brain Mapp.

[67] Awad, M., & Khanna, R. Support vector regression. in *Efficient Learning Machines: Theories, Concepts, and Applications for Engineers and System Designers*, Awad, M., & Khanna, R., Eds. Berkeley, CA: Apress, pp. 67–80. 10.1007/978-1-4302-5990-9_4 (2015).

[68] Sestieri C, Corbetta M, Romani GL, Shulman GL (2011). Episodic memory retrieval, parietal cortex, and the default mode network: Functional and topographic analyses. J. Neurosci..

[69] Cooney RE, Joormann J, Eugène F, Dennis EL, Gotlib IH (2010). Neural correlates of rumination in depression. Cognit. Affect. Behav. Neurosci..

[70] Monkul ES (2012). Abnormal resting state corticolimbic blood flow in depressed unmedicated patients with major depression: A 15O-H 2O PET study. Hum. Brain Mapp..

[71] Phillips ML, Drevets WC, Rauch SL, Lane R (2003). Neurobiology of emotion perception II: Implications for major psychiatric disorders. Biol. Psychiat..

[72] Pinel, J. P. J. Biopsychology 5th edition. *Pearson. Education* (2003).

[73] Hu H, Cui Y, Yang Y (2020). Circuits and functions of the lateral habenula in health and in disease. Nat. Rev. Neurosci..

[74] Yang Y, Wang H, Hu J, Hu H (2018). Lateral habenula in the pathophysiology of depression. Curr. Opin. Neurobiol..

[75] Browne CA, Hammack R, Lucki I (2018). Dysregulation of the lateral habenula in major depressive disorder. Front Synaptic Neurosci..

[76] Covington HE (2010). Antidepressant effect of optogenetic stimulation of the medial prefrontal cortex. J. Neurosci..

[77] Iacoboni M, Zaidel E (2004). Interhemispheric visuo-motor integration in humans: The role of the superior parietal cortex. Neuropsychologia.

[78] Ricardo-Garcell J (2009). EEG sources in a group of patients with major depressive disorders. Int. J. Psychophysiol..

[79] van der Vinne N, Vollebregt MA, van Putten MAJM, Arns M (2017). Frontal alpha asymmetry as a diagnostic marker in depression: Fact or fiction: A meta-analysis. NeuroImage Clin..

[80] Olbrich S, Tränkner A, Chittka T, Hegerl U, Schönknecht P (2014). Functional connectivity in major depression: Increased phase synchronization between frontal cortical EEG-source estimates. Psychiatry Res. Neuroimaging.

[81] Messerotti Benvenuti S, Buodo G, Mennella R, Dal Bò E, Palomba D (2019). Appetitive and aversive motivation in depression: The temporal dynamics of task-elicited asymmetries in alpha oscillations. Sci. Rep..

[82] Trambaiolli, L. R., & Biazoli, C.E. Resting-state global EEG connectivity predicts depression and anxiety severity. in *2020 42nd Annual International Conference of the IEEE Engineering in Medicine & Biology Society (EMBC)*, Montreal, QC, Canada, pp. 3707–371010.1109/EMBC44109.2020.9176161 (2020). 10.1109/EMBC44109.2020.9176161 33018806

[83] Desimone R, Duncan J (1995). Neural mechanisms of selective visual attention. Annu. Rev. Neurosci..

[84] Aftanas LI, Golocheikine SA (2001). Human anterior and frontal midline theta and lower alpha reflect emotionally positive state and internalized attention: High-resolution EEG investigation of meditation. Neurosci. Lett..

[85] Aftanas LI, Varlamov AA, Pavlov SV, Makhnev VP, Reva NV (2002). Time-dependent cortical asymmetries induced by emotional arousal: EEG analysis of event-related synchronization and desynchronization in individually defined frequency bands. Int. J. Psychophysiol..

[86] Arns M (2015). Frontal and rostral anterior cingulate (rACC) theta EEG in depression: Implications for treatment outcome?. Eur. Neuropsychopharmacol..

[87] Wang YP, Gorenstein C (2013). Assessment of depression in medical patients: A systematic review of the utility of the Beck Depression Inventory-II. Clinics.

[88] Bowling A (2005). Mode of questionnaire administration can have serious effects on data quality. J. Public Health.

[89] Moore, M. J., Moore, P. B., & Shaw, P. J. Mood disturbances in motor neurone disease. 10.1016/S0022-510X(98)00203-2 (1998). 10.1016/s0022-510x(98)00203-2 9851650

[90] Price JL, Drevets WC (2010). Neurocircuitry of mood disorders. Neuropsychopharmacology.

[91] Maj M (2011). When does depression become a mental disorder?. Br. J. Psychiatry.

[92] M. Maj (2016) The continuum of depressive states in the population and the differential diagnosis between ‘normal’ sadness and clinical depression. in *Sadness or Depression? International Perspectives on the Depression Epidemic and Its Meaning*, J. C. Wakefield and S. Demazeux, Eds. Dordrecht: Springer Netherlands, 2016, pp. 29–38. 10.1007/978-94-017-7423-9_3.

[93] Ruscio J, Ruscio AM (2000). Informing the continuity controversy: A taxometric analysis of depression. J. Abnorm. Psychol..

[94] Ing A (2019). Identification of neurobehavioural symptom groups based on shared brain mechanisms. Nat. Hum. Behav..

[95] Breiman L, Spector P (1992). Submodel selection and evaluation in regression. The X-Random Case. Int. Stat. Rev..

[96] Pereira F, Mitchell T, Botvinick M (2009). Machine learning classifiers and fMRI: A tutorial overview. Neuroimage.

[97] Varoquaux G, Raamana PR, Engemann DA, Hoyos-Idrobo A, Schwartz Y, Thirion B (2017). Assessing and tuning brain decoders: Cross-validation, caveats, and guidelines. Neuroimage.

[98] Ko SM, Kua EH, Chow MH (1997). Depression of young and elderly patients. Singapore Med. J..

