# Supplementary material for: An electroencephalography connectome predictive model of major depressive disorder severity
Source: Sci Rep. 2022 Apr 26;12:6816. doi: 10.1038/s41598-022-10949-8 (PMC9042869; doi:10.1038/s41598-022-10949-8)
Supplement: Supplementary file 1 — Supplementary Information. [file 41598_2022_10949_MOESM1_ESM.docx]

**Supplementary materials**

**An Electroencephalography connectome predictive model of major depressive disorder severity**

Aya Kabbara^1,2^, Gabriel Robert^3,4,5^, Mohamad Khalil^6,7^, Marc Verin^5^, Pascal Benquet^8^, Mahmoud Hassan^2,9^

^1^ Lebanese Association for Scientific Research, Tripoli, Lebanon

^2^ MINDig, F-35000 Rennes, France

^3^ Academic department of Psychiatry, Centre Hospitalier Guillaume Régnier, France

^4^ Empenn, U1228, IRISA, UMR 6074, Rennes, France

^5^ Comportement et noyaux gris centraux, EA 4712, CHU Rennes, Université de Rennes 1, Rennes 35000, France.

^6^ Azm Center for Research in Biotechnology and Its Applications, EDST, Tripoli, Lebanon.

^7^ CRSI research center, Faculty of Engineering, Lebanese University, Beirut, Lebanon

^8^  Univ Rennes, Inserm, LTSI - U1099, F-35000 Rennes, France

^9^ School of Science and Engineering, Reykjavik University, Reykjavik, Iceland.

Corresponding author: Mahmoud Hassan, mahmoud.hassan.work@gmail.com

|  | **HC group** | **MDD group** | **All participants** |
| --- | --- | --- | --- |
| **High and low depressive edges** | 0.3525±0.07 | 0.3921±0.04 | 0.59±0.1 |
| **High depressive edges** | 0.257±0.08 | 0.0667±0.13 | *0*.42 ±0.09 |
| **Low depressive edges** | 0.0992±0.1 | 0.4017±0.06 | *0*.61± 0.08 |

Table S1. Correlations values of the model for Dataset 1

|  | **HC group** | **MDD group** | **All participants** |
| --- | --- | --- | --- |
| **High and low depressive edges** | 0.4109 | 0.21 | 0.52 |
| **High depressive edges** | 0.2401 | 0.0391 | 0.4 |
| **Low depressive edges** | 0.182 | -0.0081 | 0.32 |

Table S2. Correlations values of the model for Dataset 2


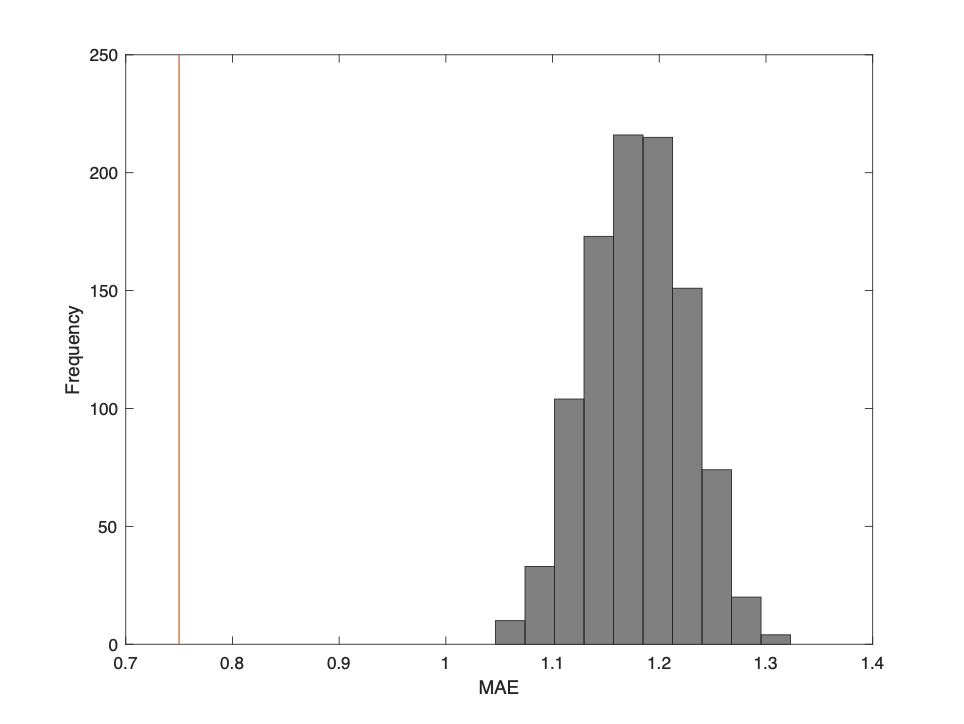


Figure S1. The MAE null distribution of the model established on high and low depressive edges in Dataset 1. The red line indicates the MAE value of the model.


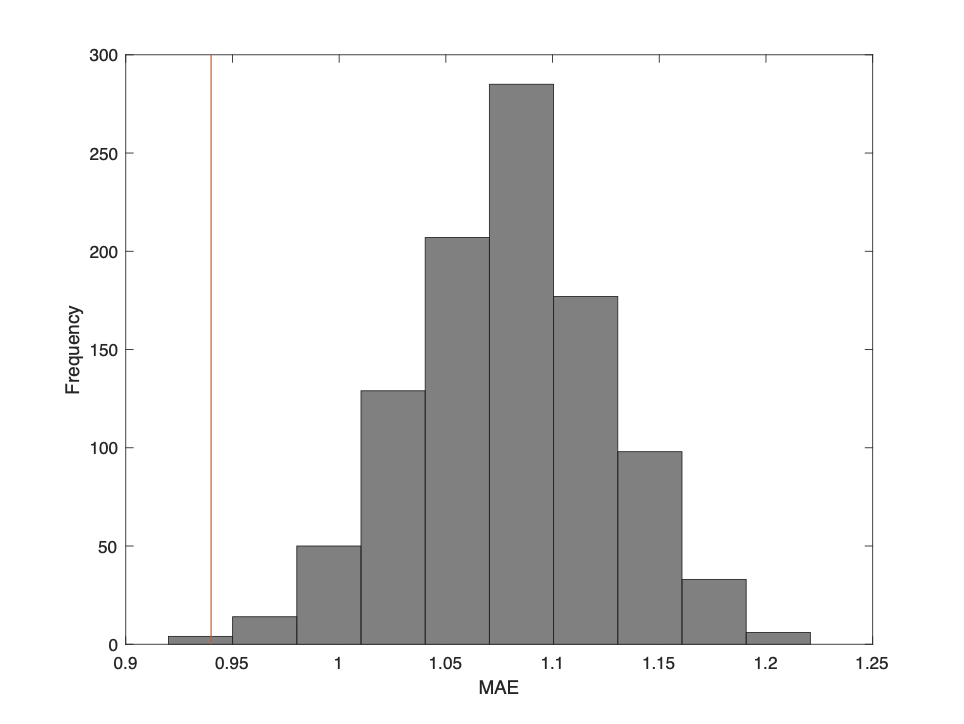


Figure S2. The MAE null distribution of the model established on high depressive edges in Dataset 1. The red line indicates the MAE value of the model.


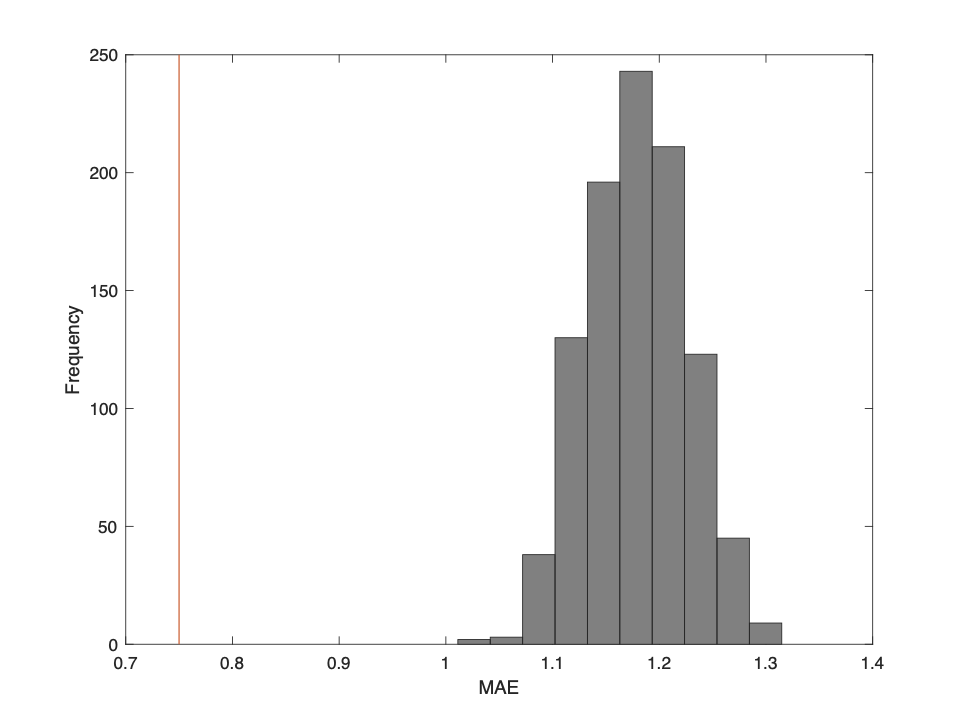


Figure S3. The MAE null distribution of the model established on low depressive edges in Dataset 1. The red line indicates the MAE value of the model.


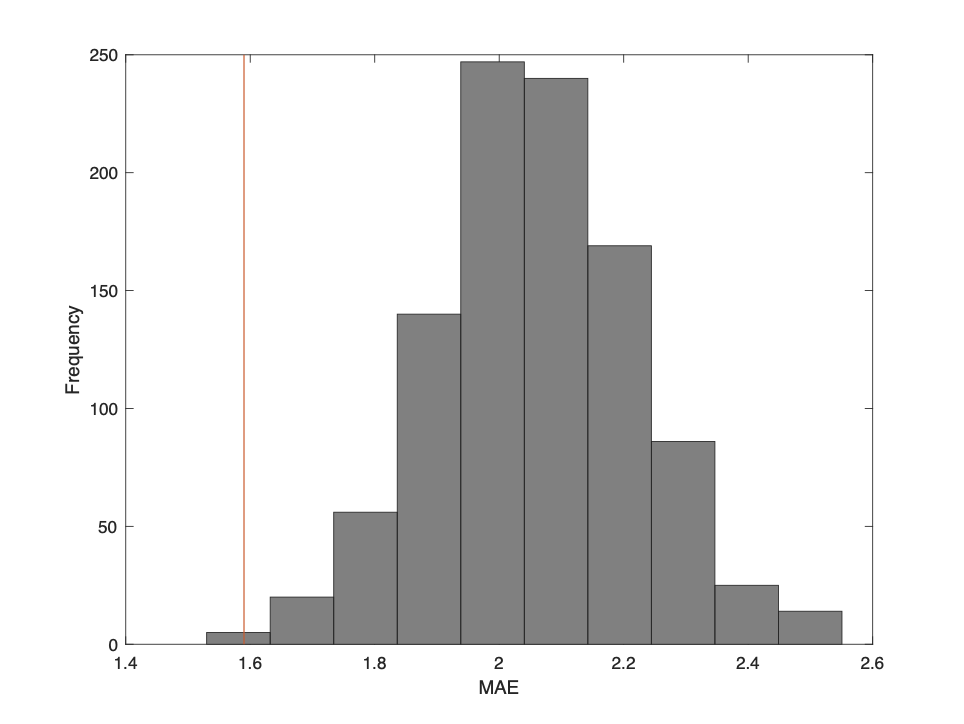


Figure S4. The MAE null distribution of the model established on high depressive edges in Dataset 2. The red line indicates the MAE value of the model.


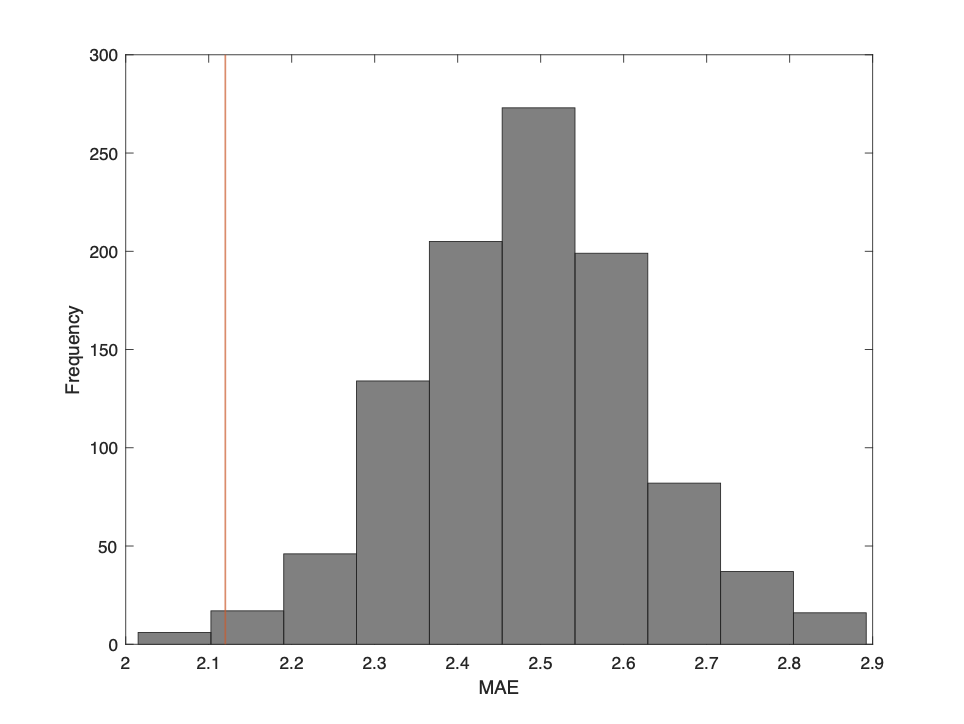


Figure S5. The MAE null distribution of the model established on low depressive edges in Dataset 2. The red line indicates the MAE value of the model.


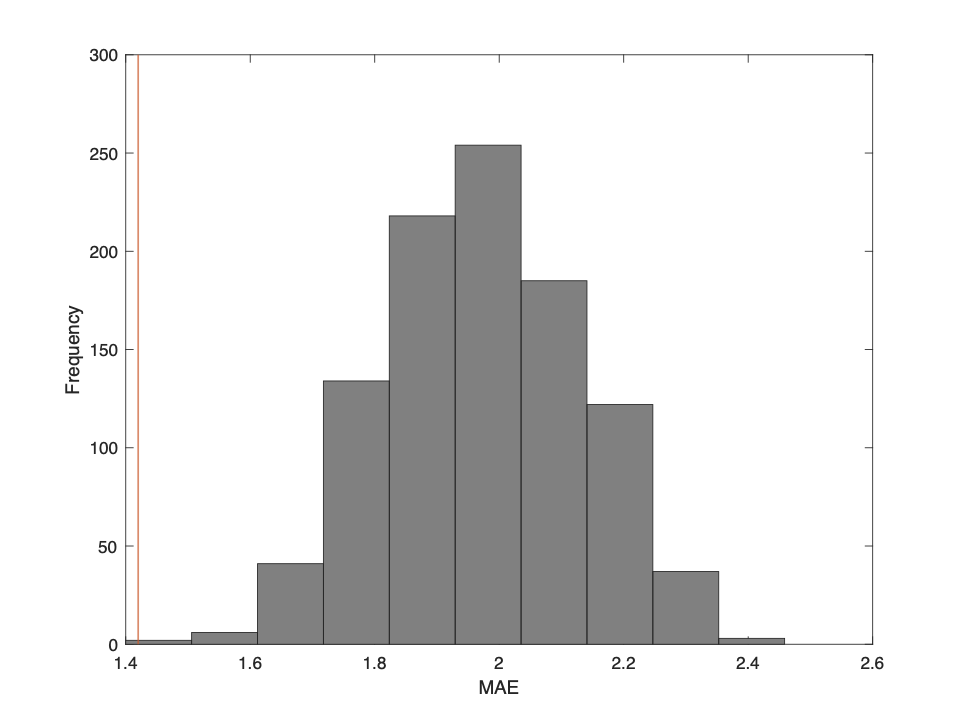


Figure S6. The MAE null distribution of the model established on high and low depressive edges in Dataset 2. The red line indicates the MAE value of the model.


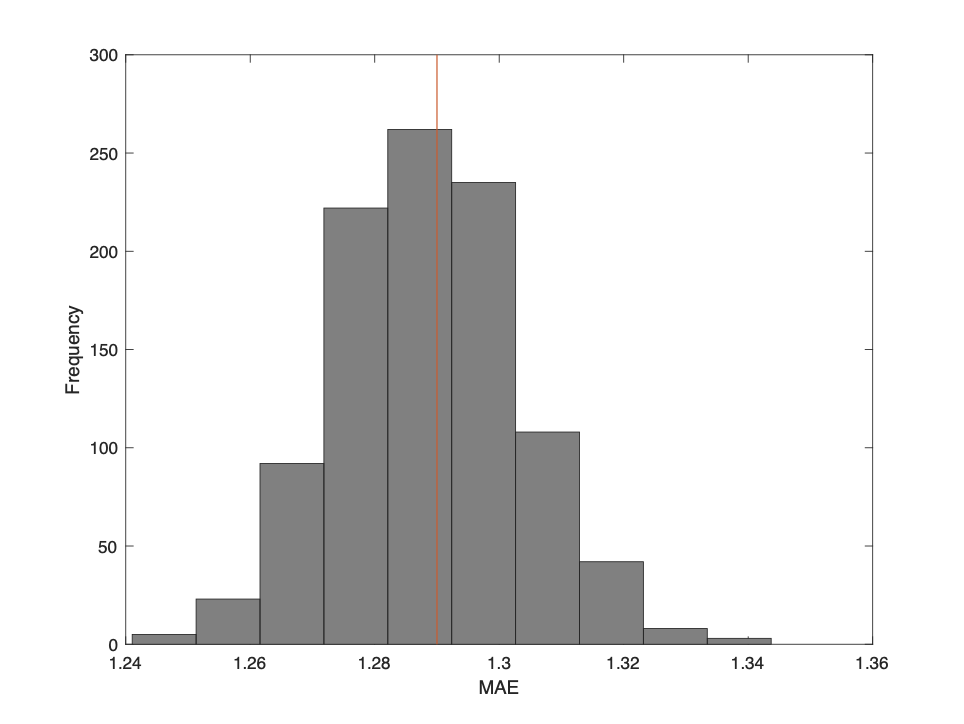


Figure S7. The MAE null distribution of the model established on low depressive edges in Dataset 3. The red line indicates the MAE value of the model.


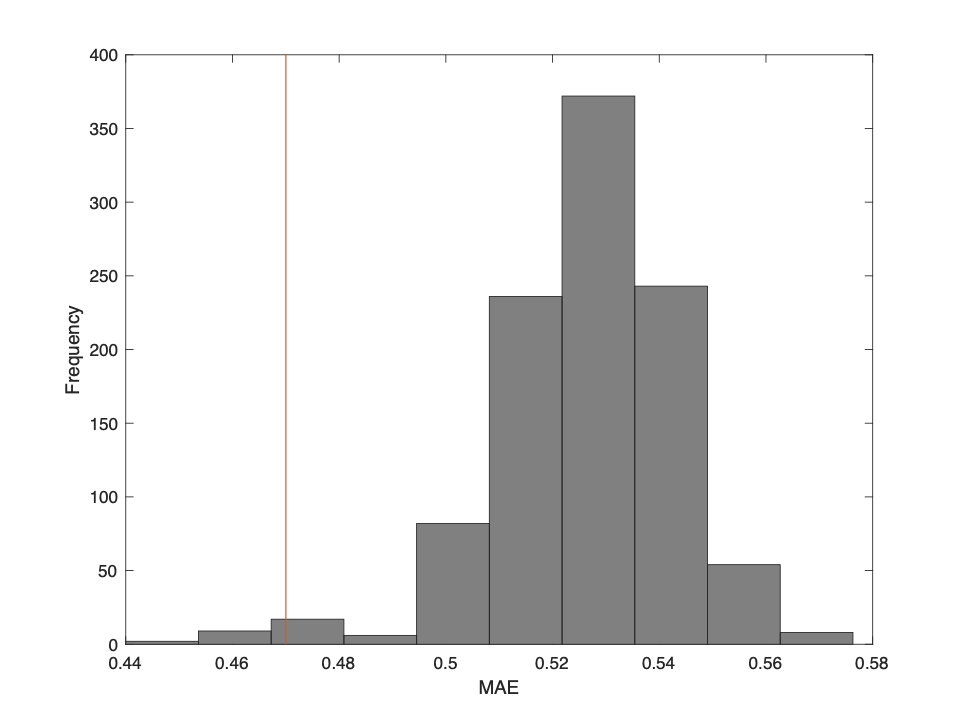


Figure S8. The MAE null distribution of the model established on high depressive edges in Dataset 3. The red line indicates the MAE value of the model.
